# Differential near-infrared imaging of heterocysts using single-walled carbon nanotubes

**DOI:** 10.1007/s43630-022-00302-3

**Published:** 2022-10-03

**Authors:** Alessandra Antonucci, Melania Reggente, Alice J. Gillen, Charlotte Roullier, Benjamin P. Lambert, Ardemis A. Boghossian

**Affiliations:** Institute of Chemical Sciences and Engineering (ISIC), Ecole Polytechnique Fédérale de Lausanne (EPFL), 1015 Lausanne, Switzerland

**Keywords:** Single-walled carbon nanotubes (SWCNTs or SWNTs), Cyanobacteria, Bioconjugation, Near-infrared (NIR) fluorescence, Cellular uptake, Lysozyme, *Synechocystis* sp. PCC 6803, *Nostoc punctiforme*

## Abstract

The internalization of near-infrared (NIR) optical nanoprobes in photosynthetic microbes can be exploited for applications ranging from energy conversion to biomolecule delivery. However, the intrinsic, species-dependent properties of microbial cell walls, including their surface charge density, composition, thickness, and elasticity, can severely impact nanoprobe uptake and affect the cellular response. An examination of the interaction of the optical nanoprobe in various species and its impact on cell viability is, therefore, imperative for the development of new imaging technologies. Herein, we extend the technology recently developed for internalizing fluorescent single-walled carbon nanotubes (SWCNTs) in prokaryotes, specifically unicellular *Synechocystis* sp. PCC 6803*,* to a filamentous cyanobacterial strain, *Nostoc punctiforme*. Using a combination of NIR fluorescence, scanning electron microscopy (SEM), and Raman spectroscopy, we investigate uptake in vegetative cells as well as differentiated heterocysts. We demonstrate a strong dependence of long-term cell integrity, activity, and viability on SWCNT surface functionalization. We further show differential uptake of SWCNTs across a single filament, with positively charged functionalized SWCNTs preferentially localizing within the heterocysts of the filament. This cell dependency of the nanoparticle internalization motivates the use of SWCNTs as a NIR stain for monitoring cell differentiation.

**Graphical abstract:**

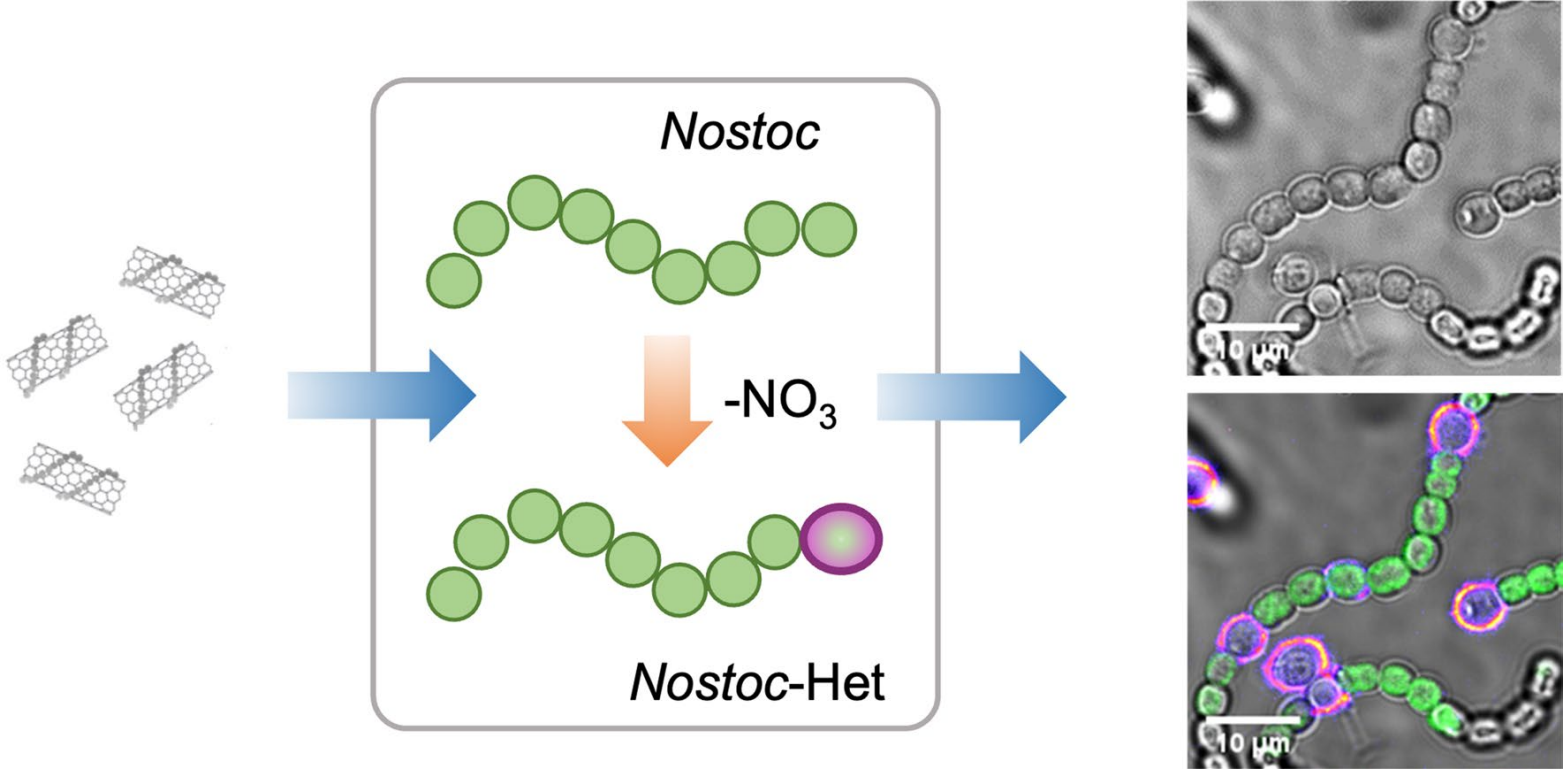

**Supplementary Information:**

The online version contains supplementary material available at 10.1007/s43630-022-00302-3.

## Introduction

Semiconducting single-walled carbon nanotubes (SWCNTs) are being used in a growing number of whole-cell technologies, including intracellular imaging and sensing, [1, 2] gene-delivery, [3] and cancer treatment. [4] The fluorescence properties of SWCNTs are practical not only for studying cellular dynamics but also for investigating nanoparticle transport. Their fluorescence emissions enable the spatiotemporal mapping of intra- and extra-cellular fluxes, [5, 6] and real-time imaging can be used to monitor SWCNT translocation across cellular membranes both in vitro and in vivo. [7–9] These applications strongly depend on the governing interactions between the SWCNTs and the living cells. Since these interactions vary with factors, such as cell type, incubation conditions, and nanotube properties [10], the translation of emerging SWCNT technologies to new cells requires a thorough investigation of the biocompatibility, specificity, and efficacy of the engineered nanoprobe in the new host.

Previous studies have largely focused on SWCNT translocation across the cell membranes of various eukaryotes. SWCNT translocation has been shown to depend on a range of physical and chemical factors, including length, diameter, surface functionalization, and charge density, along with dispersion quality. These studies have reported both active and passive uptake mechanisms [11, 12]. Although early studies showed SWCNT uptake to be independent of cell type [13], more recent findings have reported cell-specific interaction and uptake mechanisms when examining a wider range of incubation and functionalization conditions. [14, 15] Moreover, intrinsic cell membrane properties, including surface charge, composition, thickness, and elasticity, have been shown to affect the extent to which nanoparticles are ultimately able to penetrate cell compartments and tissues. [16, 17] Beyond regulating nanoparticle transport, Roxbury et al*.* have reported that cell surface electrostatic potentials, which are mediated by cell-specific membrane proteins, can also modulate the optical response of fluorescent SWCNTs. [18]

The more limited number of studies done with prokaryotic cells similarly suggest that the outer membranes play a central role in mediating the cell–nanoparticle interaction via passive uptake mechanisms. Notably, the surface charge of bacterial cell walls, which has been shown to modulate cell aggregation or biofilm formation, can dictate the efficiency of nanoparticle–cell association and subsequent internalization. [19] Most bacterial walls exhibit a net negative charge due to the presence of specific anionic components, such as lipopolysaccharides. Jacobson et al*.* showed that the structure and density of the lipopolysaccharides govern the extent and the distance of a nanoparticle’s interaction with the outer membranes of Gram-negative bacteria. [20] This interaction is largely dominated by electrostatic forces, with cationic nanoparticles prevailing over anionic materials with respect to binding to the negatively charged cell leaflets.

**Fig. 1:**
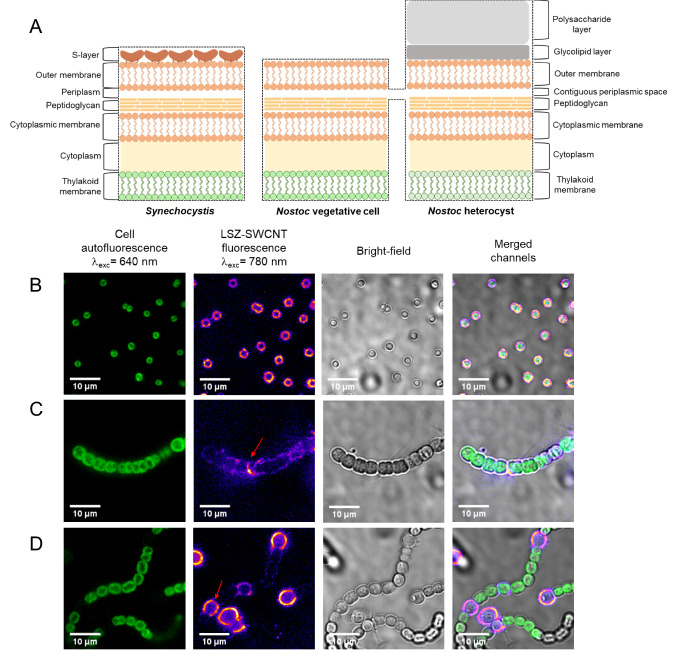
NIR Imaging of Unicellular *Synechocystis* and Filamentous *Nostoc* Cells with LSZ–SWCNTs. **A** Schematic illustration of the cell wall architecture of *Synechocystis*, vegetative *Nostoc*, and *Nostoc* heterocysts. Representative images of **B** *Synechocystis*, **C** *Nostoc*, and **D** *Nostoc*-Het cells after incubation with LSZ–SWCNTs. Fluorescence emissions were used to image cell autofluorescence (excitation at 640 nm, emission above 800 nm) and SWCNT fluorescence in confocal mode (excitation at 780 nm, emission above 980 nm). Red arrows indicate significant SWCNT fluorescence signals localizing within the peripheral regions of *Nostoc* and *Nostoc*-Het cells

In our recent study, fluorescent SWCNTs were used to image photosynthetic prokaryotes, which benefit from the NIR SWCNT fluorescence that is distinct from the cell’s autofluorescence. [21] This study showed that the zeta-potential of functionalized SWCNTs was critical for facilitating nanoparticle uptake in living *Synechocystis* sp. PCC 6803 (hereafter referred to as *Synechocystis*) cells. Like *Synechocystis*, *Nostoc punctiforme* cells (hereafter referred to as *Nostoc*) are photosynthetic prokaryotes that are surrounded by a negatively charged cell wall that is composed of inner and outer membranes that are separated by a large periplasmic space and a thick peptidoglycan layer (Fig. 1A). [22] Despite similarities to *Synechocystis*, *Nostoc* cells lack DNA competence and external surface layers, such as S-layers, which provide the cells with increased resistance against both mechanical and osmotic stress. [23] In addition, *Nostoc* cells can assemble into multicellular filaments that are distinct from the unicellular *Synechocystis* cells. Furthermore, under nitrogen deprivation, 5–10% of the vegetative cells comprising these filaments differentiate to heterocysts, which are specialized compartments for N_2_ fixation. [24] Heterocysts are characterized by a distinct pigmentation and an additional cell envelope that is composed of two chemically different layers deposited on top of the outer membrane (Fig. 1A). [25] This bilayered structure limits the permeation of gas, ions, and other hydrophilic solutes inside the heterocyst, establishing a microoxic environment that is needed for the expression and function of enzymes devoted to nitrogen fixation. [24] The distinct membrane structures in the vegetative cells and heterocysts and the multicellular assembly of the *Nostoc* filamentous strain present a largely unexplored avenue for studying nanoprobe uptake across differential cell architectures.

In this study, we explore the interaction of SWCNTs with the filamentous *Nostoc* strain. The strain’s distinct cell wall architecture, along with the heterogeneity of its cell types, its visible autofluorescence, and its inability to naturally uptake DNA, motivates the need to explore SWCNTs as both delivery and NIR imaging agents for this strain. We employ fluorescence microscopy alongside other complementary techniques, such as SEM and confocal Raman spectroscopy, to study nanoparticle uptake and to investigate the impact of the SWCNT–cell interaction on both the cell’s integrity and activity.

## Materials and methods

### Functionalization of SWCNTs

The SWCNTs used in this study were purified HiPco nanotubes (NanoIntegris, Lot. No. HP26-019) with a mean diameter of 0.8–1.2 nm and length of 100–1000 nm. LSZ–SWCNTs were prepared by suspending 1 mg of HiPco nanotubes and 5 mg of lysozyme from chicken egg white (Sigma Aldrich) in 1 mL of 1 mM HEPES buffer (pH 7.4) and sonicating using a cup-horn sonicator (140 mm, Qsonica, LLC) for 90 min at 1% amplitude on ice. Chitosan–SWCNTs were prepared as described in Reuel et al*.* [26] Briefly, 1 mg of HiPco nanotubes were suspended in 1 mL of 2.5 mg/mL chitosan (Carl Roth) solution in 1 mM HEPES buffer with 1% acetic acid. The sample was then sonicated for 90 min at 1% amplitude on ice using cup-horn sonication.

All sonicated SWCNT suspensions were centrifuged (Eppendorf Centrifuge 5424 R) for 180 min at 16500×*g* to pellet SWCNT aggregates. Unbound proteins and polymers were removed through dialysis against 2 L of 1 mM HEPES buffer using a 300 kDa MWCO cellulose membrane. SWCNT concentrations were calculated from absorbance measurements at 632 nm in a UV–Vis–NIR scanning spectrophotometer (Shimadzu 3600 Plus) using an extinction coefficient of 0.036 L/(mg cm) [27].

### Bacterial strains and growth conditions

Liquid cultures of wild-type *Synechocystis* and *Nostoc* were grown in BG11 medium (containing NaNO_3_ as the nitrogen source according to Rippka et al*.* [28]) or BG11_0_ (without addition of a nitrogen source) supplemented with 10 mM TES buffer (pH 8.0) at 30 °C under 50 µmol photons m^−2^ s^−1^ of white light with constant shaking (180 rpm).

### NIR fluorescence imaging

Cells were harvested during the mid-exponential growth phase (OD_750nm_ between 1 and 1.5), pelleted by centrifugation, washed twice with 1 mM HEPES buffer (pH 7.4), and re-suspended in the same buffer to obtain an OD_750nm_ = 0.9. Cells were fixed onto poly-L-lysine coated glass-bottom petri dishes by spotting 30 µL of the cell–SWCNT suspensions for 10 min, followed by washing with 1 mM HEPES buffer (pH 7.4). Fixed cells were treated with 50 µL LSZ–SWCNTs (concentration of 2 mg/L) and incubated at room temperature for 10 min in the dark prior to washing with 1 mM HEPES buffer (pH 7.4).

Cells were imaged using a custom-built optical setup consisting of an inverted microscope (Eclipse Ti-E, Nikon AG Instruments) with an oil-immersion TIRF Apo 100 × objective (N.A. 1.49, Nikon) coupled to a CREST X-Light spinning-disk confocal imaging system (CREST Optics) (60 µm pinholes) and an InGaAs camera (NIRvana 640 ST, Princeton Instruments). The setup has an axial resolution of 0.6 ± 0.1 μm and a lateral resolution 0.5 ± 0.1 μm. Samples were illuminated using a TriLine LaserBank system (Cairn Research) at 640 nm and 780 nm, and fluorescence was collected using either an 800 nm (Chroma) or a 980 nm long-pass filter (Semrock). Images were acquired using the Nikon NIS-Elements software (Nikon Instruments).

### Zeta-potential measurements

All stock solutions of SWCNTs were diluted in 1 mM HEPES buffer (pH 7.4) to yield a final concentration of 10 mg/L. Cell suspensions were diluted to an OD_750nm_ = 0.9. Zeta potential measurements were carried out with a Zetasizer Nano ZS analyzer (Malvern) using folded capillary cells.

### Raman characterization

Raman spectra were recorded at an excitation wavelength of 532 nm from 200 to 1800 cm^−1^ using a water-immersion 63 × objective (0.90 N.A.) on a confocal spectroscope (inVia Raman Microscope, Renishaw). For automatic confocal Raman mapping, confocal Raman spectra were recorded with a step size < 1 µm in the X–Y plane, for a total number of 9 × 9 spectra. Spectra were automatically acquired along the Z direction, where Z = 0 corresponds to the highest contrast of the cell on a bright-field image. The setup has an axial resolution of 2 μm. The spectrometer was calibrated before measurements using an internal standard. 3D maps were generated using a custom Matlab script (Matlab R2015, Mathworks).

### Scanning electron microscopy (SEM) experiments

Cells were harvested during mid-exponential growth phase, pelleted by centrifugation, washed twice with 1 mM HEPES buffer (pH 7.4), and re-suspended in the same buffer to an OD_750nm_ = 0.9. The cells were fixed onto poly-L-lysine coated glass slides (15 × 15 mm, MatTek) by spotting 30 µL of the cell–SWCNT suspension for 10 min prior to rinsing with 1 mM HEPES buffer (pH 7.4). The cells were then incubated with 2 mg/L LSZ–SWCNTs for 1 h, followed by washing with 1 mM HEPES buffer (pH 7.4). Control samples were also prepared in the absence of LSZ–SWCNTs. All samples were immersed in a 1.25% glutaraldehyde solution made in 0.1 M phosphate buffer (pH 7.4) and incubated for 2 h. Following incubation, the samples were washed three times in 0.1 M cacodylate buffer (pH 7.4). Post-fixation was performed by immersing the samples into 0.2% osmium tetroxide in 0.1 M cacodylate buffer, followed by two washing steps with deionized water. Samples were immersed in 30%, 50%, 70%, 90%, 96% and 100% alcohol–water gradients for 3 min each for dehydration. The samples were critical-point dried and covered with a 4-nm osmium coating. The samples were subsequently analyzed using an ultra-high-resolution microscope (Field Emission SEM, Zeiss Merlin) with an extra-high tension (EHT) voltage of 1.5 kV.

### Measuring net oxygen evolution

Cells were harvested during the mid-exponential growth phase (OD_750nm_ between 1 and 1.5) by centrifugation, washed with 1 mM HEPES buffer, and re-suspended in the same buffer to an OD_750nm_ = 0.9. Cell suspensions were mixed with SWCNTs to yield a suspension with a final nanotube concentration of 2 mg/L. The mixtures were incubated for 1 h in the dark. The oxygen concentration under light and dark conditions for whole cells was monitored at room temperature using a Clark-type electrode (Hansatech, OxyLab + , Norfolk). Samples were illuminated at an intensity of 100 µmol photons m^−2^ s^−1^ under white light. 10 mM sodium bicarbonate (NaHCO_3_) was added to the cell suspensions prior to oxygen measurement to provide the cells with an excess carbon source.

## Results and discussion

Figure 1 shows representative NIR confocal images of unicellular *Synechocystis* cells, filamentous *Nostoc* cells grown in standard BG11, and *Nostoc* cells grown in a nitrogen-free medium (hereafter *Nostoc*-Het) following their incubation with LSZ–SWCNTs. The overlay of the confocal SWCNT fluorescence and bright-field images of *Synechocystis* cells shows preferential SWCNT localization along the cell periphery, suggesting a heterogeneous nanoparticle distribution that is in agreement with previous results. [21] In comparison, *Nostoc* cells showed a diminished SWCNT fluorescence, with preferential nanotube localization in the exposed periphery of the vegetative cells along the filament (red arrow, Fig. 1C and Figure S1B).

In contrast to both *Synechocystis* and *Nostoc* cells, the *Nostoc*-Het cells showed significant SWCNT fluorescence within the peripheral regions of selected cells interspersed within the filament (Fig. 1D and Figure S1A). These cells are nitrogen-fixing heterocysts, which lack the autofluorescent photosynthetic pigments found in their neighboring vegetative cells. A comparison of the NIR fluorescence of isolated heterocysts and of spheroplasts from vegetative cells further confirmed preferential SWCNT accumulation within the nitrogen-fixing heterocysts (Figure S2).

**Fig. 2:**
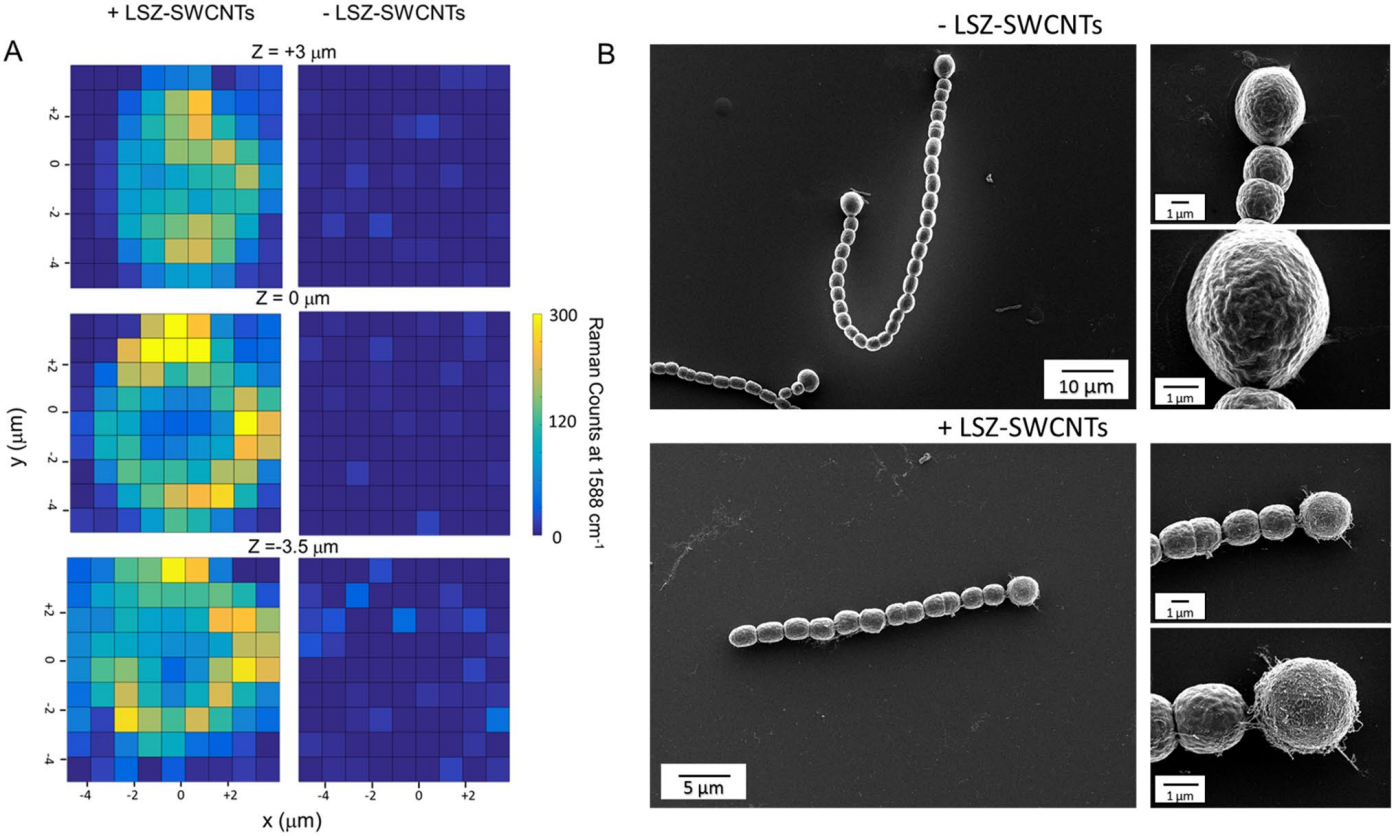
Raman and SEM Imaging of *Nostoc* Cells. **A** Representative confocal 3D Raman mapping of the characteristic G’-band (at 1580 cm^−1^) of LSZ–SWCNTs in *Nostoc-*Het under 532 nm laser excitation. Images are shown for both a terminal heterocyst from a *Nostoc* filament treated with LSZ–SWCNTs (left) and a non-treated heterocyst (right). Scans were performed at different heights within the cells, with a height of Z = 0 µm corresponding to the focal plane of the cell exhibiting the highest image contrast in the bright-field imaging mode. **B** Representative SEM images of *Nostoc* cells containing heterocysts, before (top) and after (bottom) treatment with 2 mg/L LSZ–SWCNTs

The intracellular distribution of LSZ–SWCNTs inside *Nostoc*-Het was further studied using confocal Raman spectroscopy (Fig. 2A). Confocal Z-scan maps of the characteristic G-band at 1588 cm^−1^ showed that the SWCNT signal was heterogeneously distributed throughout the volume of the nitrogen-fixing cell. In agreement with the confocal fluorescence images (Fig. 1), a higher intensity SWCNT signal was detected at the cell periphery (Fig. 2A, Figures S4 and S5). Conversely, weak SWCNT Raman signals were observed within the vegetative cells along the same filament, confirming the preferential accumulation of SWCNTs within the nitrogen-fixing compartment of the filament (Figures S4 and S5).

The extracellular distribution of the LSZ–SWCNTs was studied using scanning electron microscopy (SEM). Representative SEM images (Fig. 2B) showed that untreated vegetative *Nostoc* and *Nostoc*-Het cells possessed undisrupted, smooth outer membrane surfaces in the absence of nanotubes. Following nanoparticle exposure, the heterocyst cell wall appeared to become covered by an extracellular, nano-filamentous network (Fig. 2B and Figure S6). We hypothesize that this network is composed of polysaccharide membrane fragments as well as possible membrane-associated protein-coated nanotubes. [7] In addition, we observed significant distortions in the cell walls of vegetative *Nostoc* cells along the filament, which may affect cell viability (Figure S6). Control SEM images of individual LSZ–SWCNTs immobilized onto poly-L-lysine coated glass coverslips showed tubular structures resembling those observed on the exterior of the heterocyst (Figure S7). These observations further suggest that the nanotubes protrude from the cell surface.

The cell-specific localization of the SWCNTs is attributed to the distinct cell wall architectures of *Synechocystis*, vegetative *Nostoc*, and *Nostoc*-Het cells (Fig. 1A). Previous studies have proposed internalization mechanisms based on both membrane piercing of high-aspect nanomaterials [11] as well as charge interactions with the polymer wrapping [10]. These considerations indicate a strong dependence of the extent of the uptake on the cell wall composition. Such variations in cell wall composition, especially with regard to the density of negatively charged macromolecules, such as lipopolysaccharides, are known to strongly impact cell–nanoparticle interactions. For example, Jacobson et al*.* have demonstrated a 70% decrease in the number of Gram-negative bacterial cells with associated positively charged gold nanoparticles following the removal of 50% of the cell’s lypopolysaccharide content. [20] The *Synechocystis* cells used in the study herein are surrounded by external surface layers, or S-layers, consisting of two-dimensional crystalline arrays of proteinaceous subunits that cover the entire surface of the cells (Fig. 1A). These units are composed of weakly acidic glycoproteins that contain 40–60% hydrophobic amino acids and possess an average isoelectric point of 4–6, contributing to the overall negative charge of the cells [29]. Compared to the *Synechocystis* cells, the heterocysts of the differentiated *Nostoc*-Het strain show a similar cell wall architecture, except for a bi-layered structure that lies on top of the outer membrane in lieu of the S-layer. This bilayer consists of an inner layer of hydroxylated glycolipids and an outer layer of polysaccharides (Fig. 1A). In contrast to both *Synechocystis* and *Nostoc*-Het cells, vegetative *Nostoc* cells possess neither an additional S-layer nor an additional bilayer. [22, 30] The distinct wall architectures of these cells are, therefore, expected to vary in the total amount of polysaccharide, which would alter the density of the ionic surface charge, and consequently the zeta potential, of the cell. [33]

**Fig. 3:**
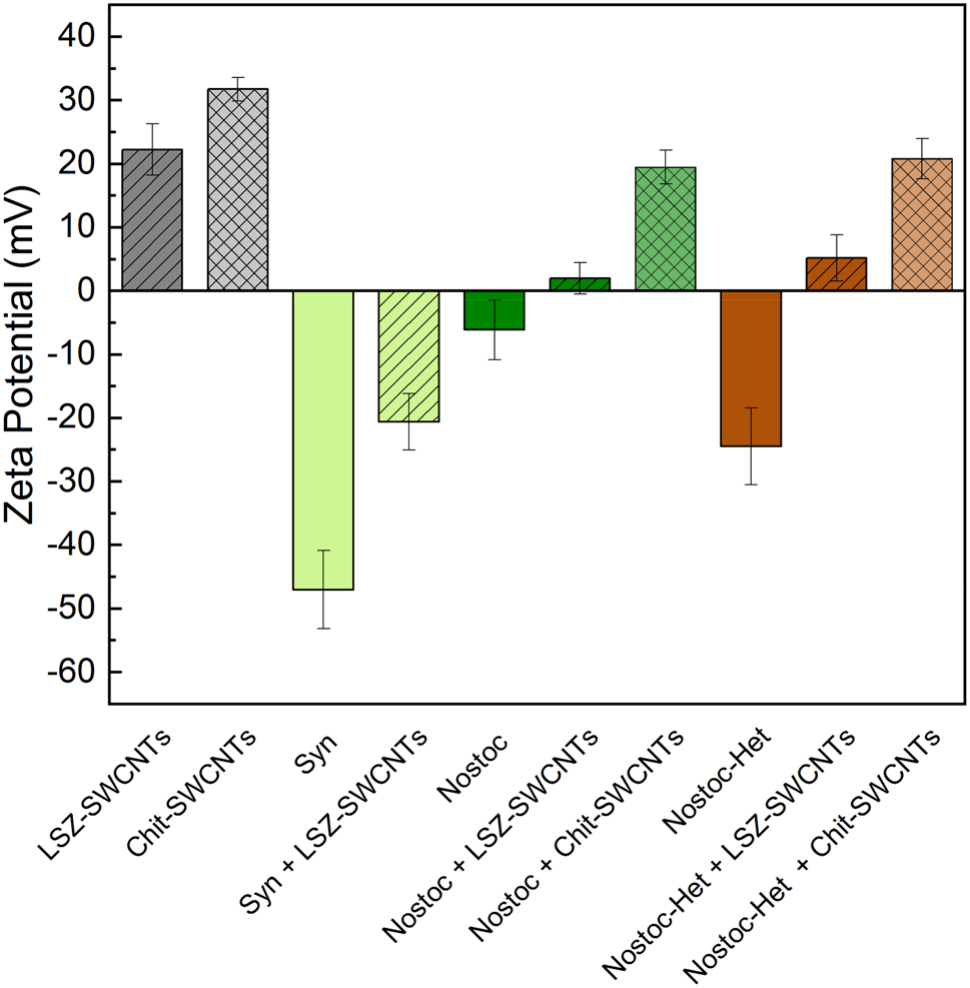
Impact of Whole Cell Zeta-Potential on SWCNT Interaction. Zeta-potentials of SWCNT-modified *Synechocystis* cells (“Syn”), *Nostoc* cells grown in nitrogen-free medium (“Nostoc-Het”), or *Nostoc* cells (“Nostoc”) grown in normal medium. Measurements were performed before and after the addition of 2 mg/L of either LSZ–SWCNTs or chitosan–SWCNTs (“Chit-SWCNTs”). Control measurements of LSZ–SWCNTs and chitosan–SWCNTs in the absence of cells are also shown on the left two bars of the figure. All samples are suspended in 1 mM HEPES buffer at pH 7.4. Error bars represent the standard deviation of three replicates

We therefore explored the correlation and effect of SWCNT uptake on the zeta potentials of the whole cells. These potentials are expected to play a critical role in modulating the electrostatic forces that govern the nanoparticle interactions. [21, 31, 32]. As shown in Fig. 3, all three cell types showed an overall negative charge at pH 7.4, with values of -47.0 ± 6.2 mV, -24.4 ± 6.1 mV, and -6.1 ± 4.7 mV for *Synechocystis*, *Nostoc*-Het and *Nostoc*, respectively. The more negative zeta-potentials of *Synechocystis* and *Nostoc*-Het cells is attributed to the higher density of negatively charged glycoproteins or polysaccharides, respectively, on the outer cell surface. The LSZ–SWCNTs, on the other hand, showed an overall positive zeta-potential that is in agreement with our previous findings [21]. The electrostatic interactions between the positively charged nanoparticles and negatively charged cells is therefore expected to alter the overall zeta potential of the whole cells. Indeed, we observed an overall decrease in the magnitude of the zeta potential for all the negatively charged cells following incubation with the LSZ–SWCNTs. In particular, the cells with a more negative initial zeta potential (~ 25 mV for *Synechocystis* and ~ 30 mV for *Nostoc-*Het) were found to undergo greater changes in their zeta potentials upon nanotube incubation. These trends are in agreement with the fluorescence images (Fig. 1B, D), which suggest increased nanoparticle interaction, and consequently a larger change in surface charge, with the more negatively charged cells. These observations thus attribute the preferential internalization of LSZ–SWCNTs in the heterocysts (Fig. 1D), at least partially, to the higher surface charge density of their cell walls.

The variable nanoparticle uptake observed for the different cell types may consequently affect their relative viability. The impact of the LSZ–SWCNTs on cell viability was therefore studied using oxygen evolution measurements as a means of monitoring the photosynthetic activity of the cells. As shown in Table 1, *Synechocystis* cells incubated in the presence of LSZ–SWCNTs exhibited similar oxygen evolution rates compared to untreated cells under the tested conditions. In contrast to the *Synechocystis* cells, both the *Nostoc* and *Nostoc*-Het cells showed a remarkable decrease in oxygen evolution rates on exposure to LSZ–SWCNTs, indicating a significant loss in cell viability. We attributed this loss in viability to either the (1) structural destabilization of the outer membrane and/or (2) the enzymatic activity of the lysozyme protein. Since structural destabilization is a primary mechanism of toxicity for cationic nanomaterials in prokaryotic cells, [34] the interaction of the positively charged LSZ–SWCNTs with *Nostoc* vegetative cells could dramatically affect their viability by altering their permeability. Similarly, the enzymatic activity of lysozyme, which is largely preserved on the nanotube surface [35], may also affect cell viability through enzymatic disruption of the cell wall. Indeed, as shown in Figure S8a, b, we observed diminished photosynthetic oxygenic evolution from *Nostoc* and *Nostoc*-Het cells treated with lysozyme either in presence or absence of SWCNTs. Compared to the *Nostoc* and *Nostoc*-Het cells, the *Synechocystis* cells are surrounded by a protective S-layer that limits the diffusion of the lysozyme to the peptidoglycan layer [36] (Fig. 1A). The absence of any additional exo-polysaccharides on the surface of the *Nostoc* vegetative cells may thus facilitate the access of lysozyme to the peptidoglycan layer, leading to the cells’ diminished viability in the presence of LSZ–SWCNTs (Table 1). In agreement with this hypothesis, we observe an almost complete rupturing of the *Nostoc* filaments into individual cells following 1 h incubation (i.e., the same incubation time used for the oxygen evolution measurements) with LSZ–SWCNTs (Figure S8c).

**Table 1:** Effect of LSZ–SWCNTs on Oxygen Evolution

Oxygen evolution rates (µmol O_2_/(mgChl x h))		
Strain	Control	+ LSZ–SWCNTs
*Synechocystis*	75.6 ± 0.6	71.7 ± 7.3
*Nostoc*	81.2 ± 4.0	−0.6 ± 2.2
*Nostoc*-Het	60.1 ± 7.8	2.4 ± 4.0

Rates of oxygen evolution under illumination (100 μmol photons m^−2^ s^−1^) for treated and untreated cells of *Synechocystis*, *Nostoc,* and *Nostoc*-Het. Values are based on independent measurements obtained from three replicates and normalized by chlorophyll pigment content. The treated cells were incubated with 2 mg/L LSZ–SWCNTs

To discern the underlying mechanism of the cell deactivation, we investigated the effect of a SWCNT wrapping that lacks muramidase activity on cell viability. In particular, we examined the interaction of *Nostoc* and *Nostoc*-Het cells with chitosan, a positively charged polysaccharide that is soluble in weakly acidic solutions. Previous studies have shown that chitosan-coated SWCNTs can cross the lipid bilayer of isolated chloroplasts [10] and can be used as molecular scaffolds for the delivery of genetic material into plant cells [37]. Since the charge density of this biopolymer is strongly dependent on the pH and the degree of deacetylation, the uptake of nanoparticles suspended with this biopolymer can be affected by the preparation and incubation conditions of the cells. The study herein thus examined the uptake of 2 mg/L chitosan–SWCNTs with a measured zeta-potential = 31.7 ± 1.9 mV (Fig. 3) that would favor electrostatic uptake.

**Fig. 4:**
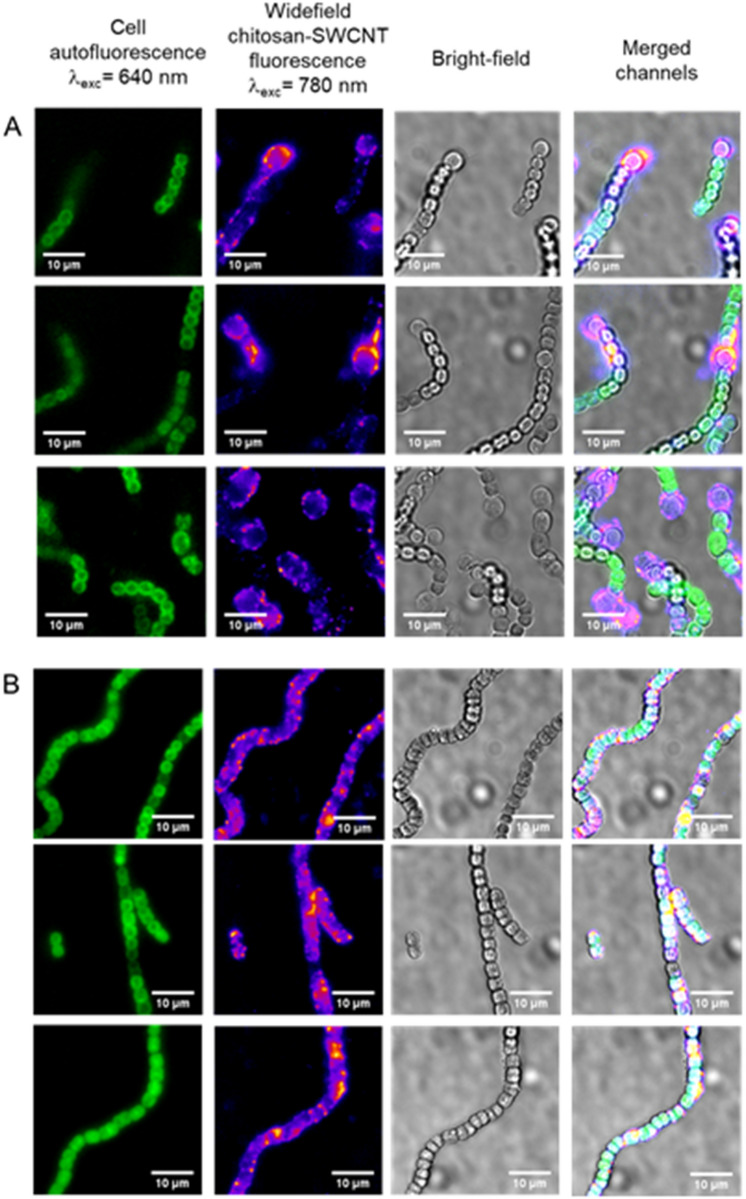
NIR Imaging of Chitosan–SWCNT Interaction with *Nostoc* Cells. Representative images of **A** *Nostoc*-Het cells and **B** *Nostoc* cells after incubation with 2 mg/L chitosan-wrapped SWCNTs for 10 min. Fluorescence intensity was recorded for cell autofluorescence (excitation at 640 nm, emission above 800 nm) and SWCNTs (excitation at 780 nm, emission above 980 nm) in widefield mode

In line with our observations for the LSZ–SWCNTs and with previous observations on chitosan uptake in plant organelles, [10, 37], the NIR fluorescence images confirmed the co-localization of chitosan–SWCNTs with the *Nostoc* filaments (Fig. 4). Due to the lower fluorescence intensities of chitosan–SWCNTs compared to LSZ–SWCNTs (Figure S9), these cell–nanoparticle interactions were monitored by NIR widefield fluorescence microscopy rather than confocal microscopy. Nonetheless, we were able to observe the same preferential localization of the chitosan–SWCNTs within the peripheral regions of heterocysts as was observed for the LSZ–SWCNTs in the confocal images. In the absence of the heterocysts, the fluorescence signals were distributed more evenly throughout the volume of *Nostoc* filaments. This uniform distribution suggests nanoparticle internalization within the cell cytoplasm. To validate this hypothesis, we monitored the fluorescence signal upon addition of potassium ferricyanide (K_3_Fe(CN)_6_), a compound capable of entering the cell periplasm and quenching SWCNT fluorescence while remaining inaccessible to the cytosol of the cell [38–40]. We observed that the fluorescence signal in the center of *Nostoc* cells incubated with chitosan–SWCNTs is largely retained (Figure S10), indicating SWCNT localization within the inaccessible compartments of the cell. This retention in fluorescence was observed for both short (10 min) and long (60 min) SWCNT incubation times. The greater uniformity in the initial fluorescence distribution for longer incubation times is attributed to increased transport of SWCNTs from the periphery towards the center of the cell over time. This observation further supports the internalization of the SWCNTs to the inner compartments.

The viability of the cells in the presence of chitosan–SWCNTs was further studied by monitoring oxygen evolution rates before and after incubation (Table 2). Under the tested conditions, both the *Nostoc* and *Nostoc*-Het cells still showed diminished photosynthetic activity compared to the *Synechocystis* cells from Table 1 following 1 h incubation with chitosan–SWCNTs. However, these cells showed significantly greater retention of activity compared to LSZ–SWCNTs. These observations suggest the viability can be engineered, at least partially, by altering the wrapping characteristics.

**Table 2:** Effect of Chitosan–SWCNTs on the Oxygen Evolution of Filamentous Cyanobacteria

Oxygen evolution rates (µmol O_2_/ (mgChl x h))		
Strain	Control	+ chitosan–SWCNTs
*Nostoc*	81.2 ± 4.0	49.1 ± 19.2
*Nostoc*-Het	60.1 ± 7.8	49.2 ± 13.3

Rates of oxygen evolution under illumination (100 µmol photons m^−2^ s^−1^) for treated and untreated cells of *Nostoc* and *Nostoc*-Het. Values are based on independent measurements obtained from three replicates and normalized by chlorophyll pigment content. The treated cells were incubated with 2 mg/L chitosan-SWCNTs

## Conclusions

In this study, we used confocal fluorescence microscopy alongside other complementary techniques, such as SEM and confocal Raman spectroscopy, to study the interaction of functionalized SWCNTs with the cells of different cyanobacterial strains. We observed that both the structure and composition of the cell wall have a profound impact on the cellular response to nanoparticle exposure. In particular, the presence of additional negatively charged layers on the outer membrane of cyanobacterial cells, such as those found in *Synechocystis* cells or *Nostoc* heterocysts, were shown to increase cell association with positively charged SWCNTs. The cytotoxicity of the nanoparticles was further shown to vary with both the cell type and wrapping, with greater viability observed for *Synechocystis* cells that are surrounded by a protective S-layer and with wrappings that lack muramidase activity. The varying susceptibility of different microbial strains to nanoparticle treatment exemplifies the complexity of these systems and motivates a systematic exploration of these strains under a wider range of conditions.

The heterocysts’ preferential uptake of both the LSZ–SWCNTs and chitosan–SWCNTs observed in this study motivates the application of these nanoparticle conjugates for monitoring cell differentiation. Compared to poly-cationic dyes (Alcian blue [41]) conventionally used to distinguish vegetative and non-vegetative cells (see Figures S11–12), the SWCNTs used in this work provide a fluorescence signal that is more clearly distinguishable between the cells. The increased photostability and the intrinsic NIR fluorescence make these SWCNT conjugates especially attractive for use in long-term imaging applications that are limited by visible dyes that have overlapping absorption and fluorescence with the cell. Furthermore, the modularity of these nanoparticle conjugates, both with regard to their surface chemistry and their differential effects of different cell wall architectures, provides a powerful means of tailoring these probes for an even broader range of applications.

## Supplementary Information

Below is the link to the electronic supplementary material.

Supplementary file1 (DOCX 14724 KB)
