# Supplementary material for: Differential near-infrared imaging of heterocysts using single-walled carbon nanotubes
Source: Photochem Photobiol Sci. 2022 Oct 3;22(1):103–13. doi: 10.1007/s43630-022-00302-3 (PMC9829582; doi:10.1007/s43630-022-00302-3)
Supplement: Supplementary file 1 — Supplementary file1 (DOCX 14724 KB) [file 43630_2022_302_MOESM1_ESM.docx]

Differential near-infrared imaging of heterocysts using single-walled carbon nanotubes

Alessandra Antonucci^a^, Melania Reggente^a^, Alice J. Gillen^a^, Charlotte Roullier^a^, Benjamin P. Lambert^a^, and Ardemis A. Boghossian^a^*

^a^Institute of Chemical Sciences and Engineering (ISIC), Ecole Polytechnique Fédérale de Lausanne (EPFL), 1015-Lausanne, Switzerland

^*^ardemis.boghossian@epfl.ch

KEYWORDS: single-walled carbon nanotubes (SWCNTs or SWNTs), cyanobacteria, bioconjugation, near-infrared (NIR) fluorescence, cellular uptake, lysozyme, *Synechocystis* sp. PCC 6803, *Nostoc sp.*

**Cell treatment with LSZ-SWCNTs in solution.** Cells were harvested during the mid-exponential growth phase (OD_750 nm_ between 1-1.5), pelleted by centrifugation, washed twice with 1 mM HEPES buffer (pH 7.4), and re-suspended in the same buffer to an OD_750 nm_ = 0.9. LSZ-SWCNTs were added to the cell suspension to a desired final concentration of 2 mg/L. Cells and SWCNTs were allowed to interact at room temperature in the dark for 10 minutes while mixing.

**Isolation of heterocysts.** Heterocysts of *Nostoc* sp. were isolated following a modified protocol from Ermakova et al. [*]

Briefly, cells were cultivated in BG11_0_ (without addition of combined nitrogen) supplemented with 10 mM TES buffer (pH 8.0) at 30°C under 50 μmol photons m^-2^ s^-1^ of white light with constant shaking. Filaments (20 mL) were harvested during the logarithmic growth phase (OD_750nm_ between 1-1.5) and pelleted by centrifugation at 5000 rpm at room temperature for 5 minutes. After discarding the growth medium, pellets were resuspended in 20 mL of extraction buffer containing 50 mM HEPES-NaOH (pH 7.2), 0.4 M sucrose, 10 mM NaCl, and 10 mM EDTA (all reagents were from Sigma Aldrich). Fresh lysozyme was added to the suspension to a final concentration of 1 mg/mL, followed by incubation for 1 hour at 30°C with continuous mixing on an orbital shaker at 180 rpm.

Cells were sonicated for 1 min in a water bath (37 kHz at 40% power) filled with cold water. After the digestion of the vegetative cells, the cell suspension was centrifuged at 1000 x g for 5 min at 4°C, and the blue supernatant was discarded. The pellet containing heterocysts was resuspended in 10 mL of cold extraction buffer, vortexed, and then centrifuged at 250 x g for 3 min at 4°C. These last steps were repeated twice until a colorless supernatant was obtained. The residual pellet was finally washed with 1 mM HEPES buffer (pH 7.4).

**Spheroplast induction in *Nostoc*.** Filaments of *Nostoc* sp. were harvested during the logarithmic growth phase (OD_750nm_ between 1-1.5) by centrifugation at 5000 rpm at room temperature for 5 minutes. The pellets were resuspended in a spheroplast-induction solution containing 0.5 M mannitol, 1 mM EDTA, 10 mM TES, and 1 mg/mL lysozyme. The cells were incubated at 30°C for 4 hours while shaking and pelleted by centrifugation at 5000 rpm for 5 min. The sedimented spheroplasts were resuspended in 1 mM HEPES buffer (pH 7.4).

**Alcian Blue staining**. The polysaccharide layer of heterocysts was stained with Alcian blue. Specifically, 2 μL of Alcian blue solution (1% in 3% acetic acid) was added to 1 mL of a culture containing *Nostoc* filaments and incubated for 10-15 minutes.

**Visible imaging.** Bright-field images were acquired using a custom-built optical setup consisting of an inverted microscope (Eclipse Ti-E, Nikon AG Instruments) with an oil-immersion TIRF Apo 100 x objective (N.A. 1.49, Nikon) coupled to a spectrometer (Shamrock 303i, Andor) operated in mirror mode (flat mirror, Al+MgF_2_ protected) and an EMCCD camera (iXon Ultra 888, Andor). Samples were illuminated with a halogen lamp. The colored images were reconstructed from three separate images acquired using an excitation light filtered with red (600 nm, Hoya R60), green (533 nm, Hoya G533), and blue (440 nm, Hoya B440) filters.


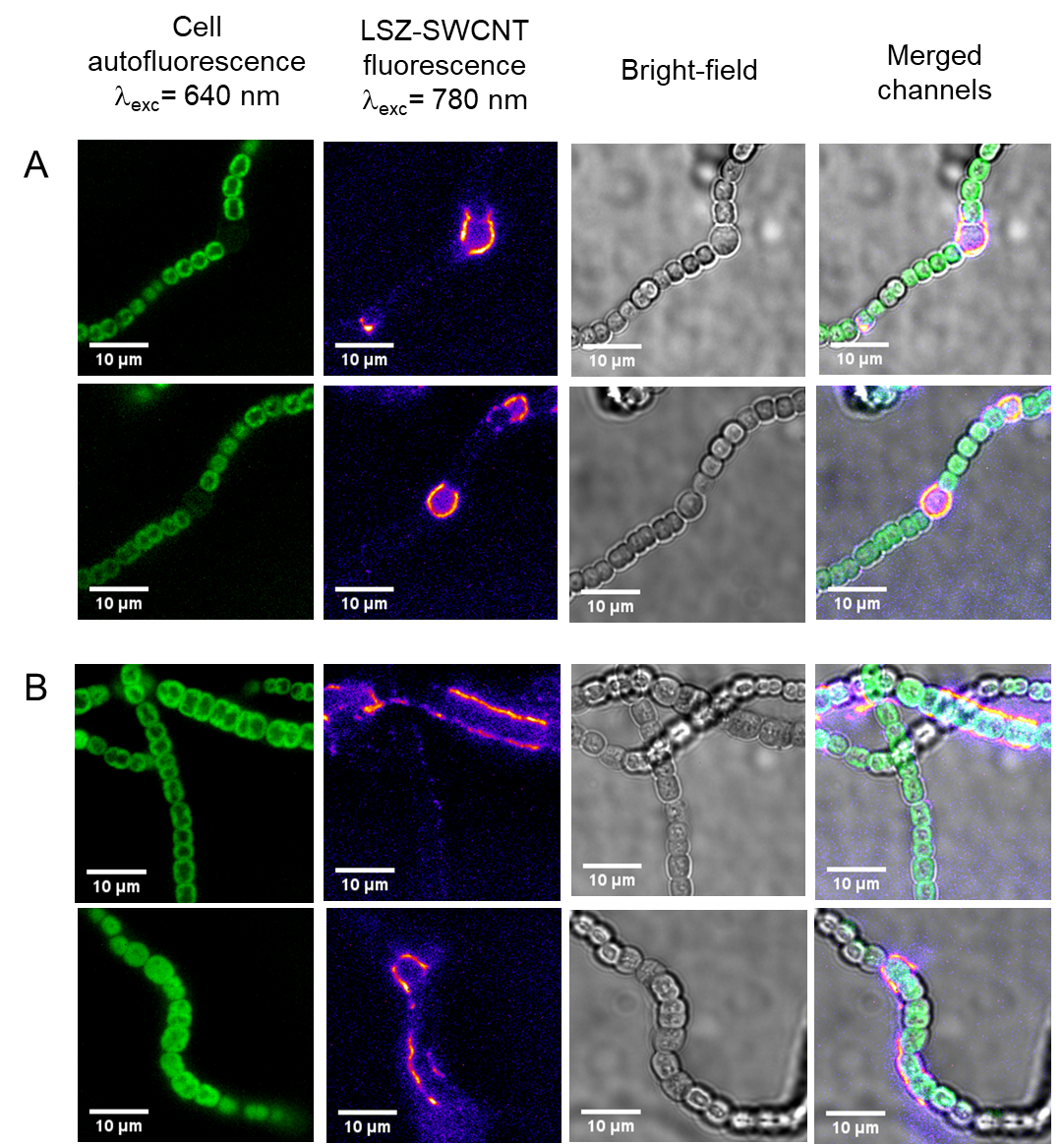


**Figure S1. NIR Imaging of LSZ-SWCNT Interaction with *Nostoc* and *Nostoc*-Het Cells.** Representative images of (A) *Nostoc*-Het cells and (B) *Nostoc* cells immobilized onto poly-lysine-coated glass slides after incubation with LSZ-wrapped SWCNTs. Fluorescence intensity was recorded for cell autofluorescence (excitation at 640 nm, emission above 800 nm) and SWCNTs in confocal mode (excitation at 780 nm, emission above 980 nm).


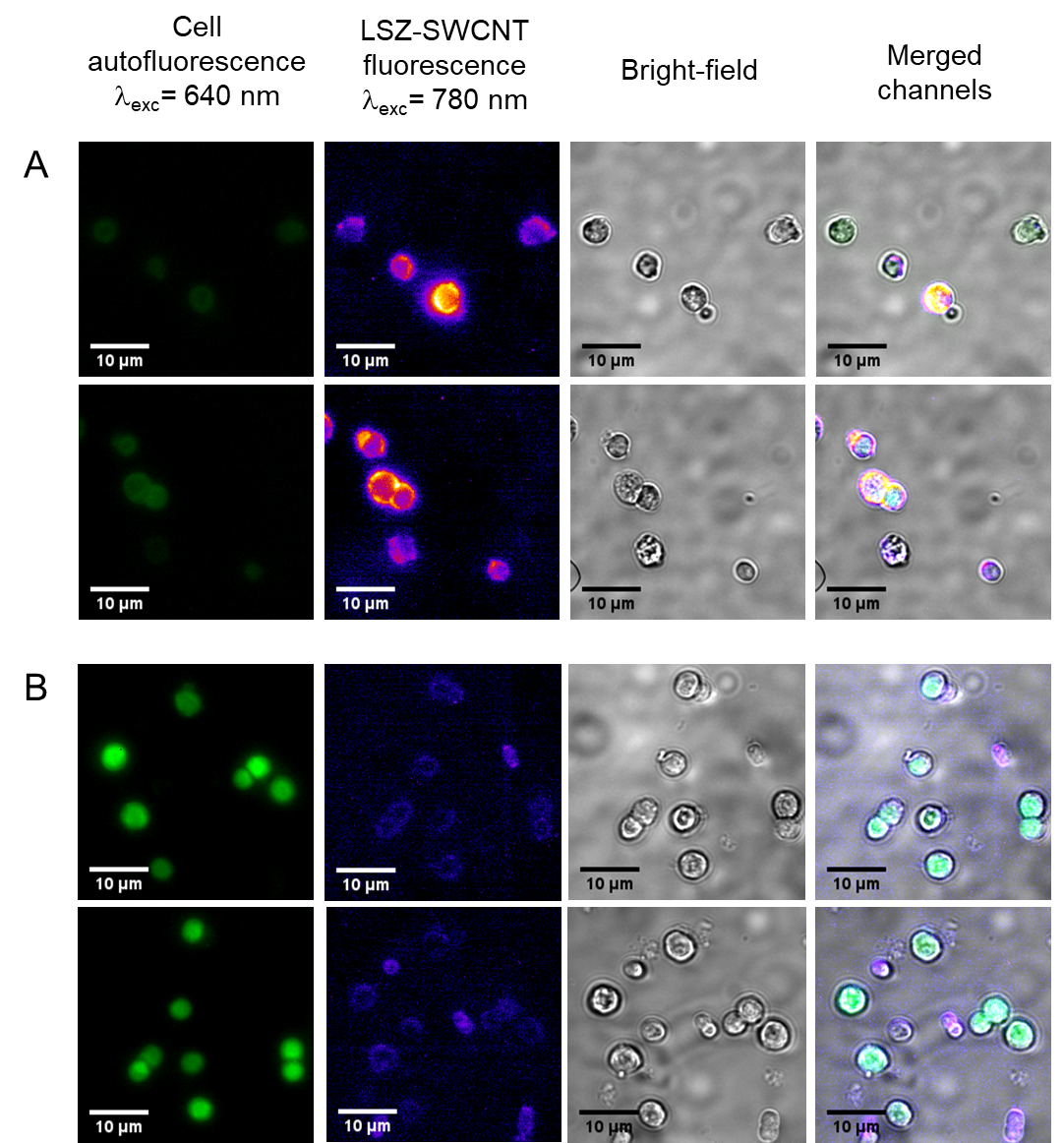


**Figure S2. NIR Imaging of SWCNT Interaction with Isolated *Nostoc* Heterocysts and Spheroplasts.** Representative images of isolated (A) *Nostoc* heterocysts and (B) *Nostoc* spheroplasts after incubation with LSZ-wrapped SWCNTs. Fluorescence intensity was recorded for cell autofluorescence (excitation at 640 nm, emission above 800 nm) and SWCNTs in confocal mode (excitation at 780 nm, emission above 980 nm). Due to diminished pigmentation, isolated heterocysts showed reduced cell autofluorescence compared to the spheroplasts of vegetative cells. By contrast, heterocysts exhibited higher SWCNT fluorescence intensity under excitation at 780 nm.


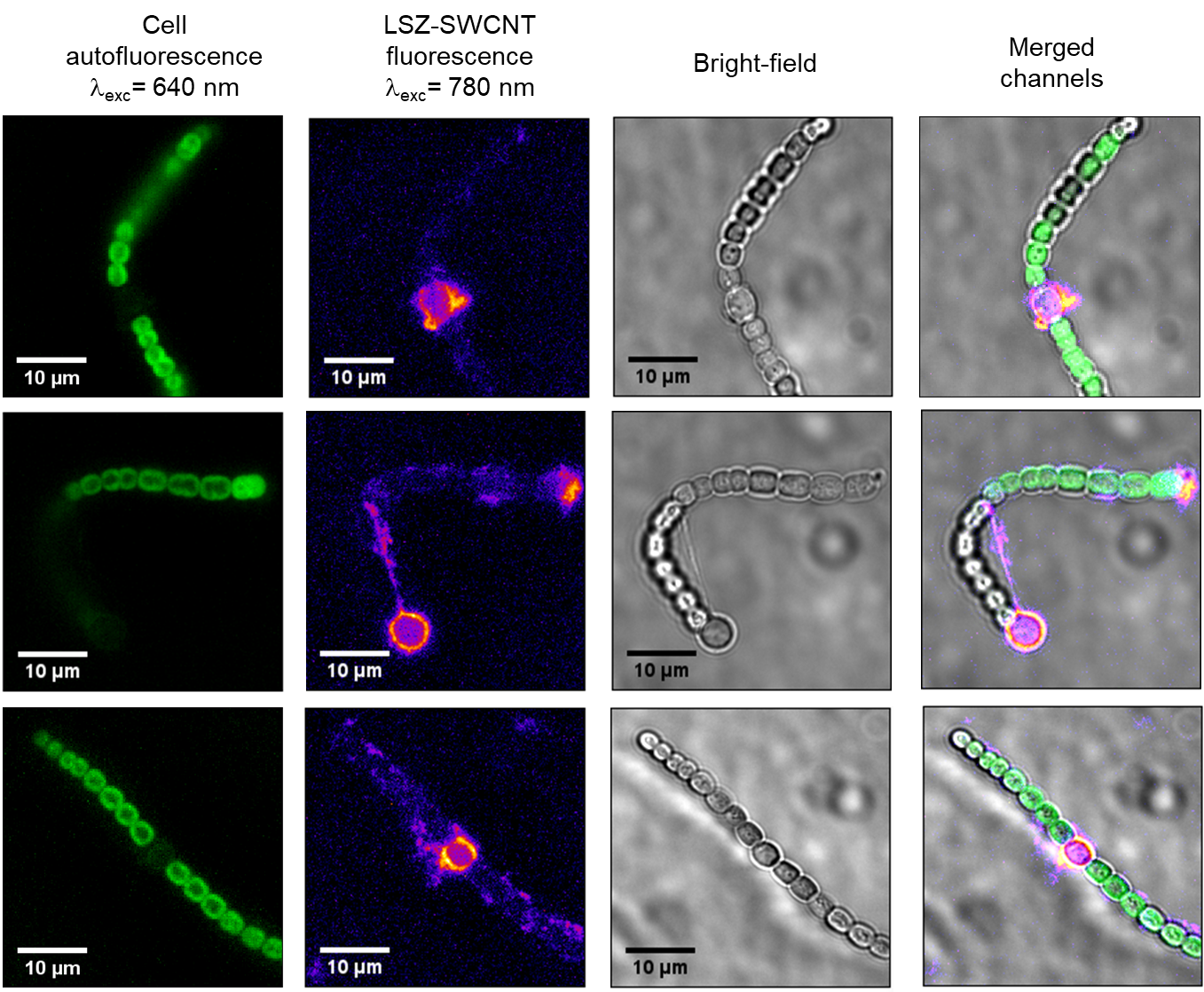


**Figure S3. NIR Imaging of SWCNT Interaction with *Nostoc*-Het Cells in Solution Under Mixing.** Representative images of *Nostoc*-Het cells after incubation with LSZ-wrapped SWCNTs in 1 mM HEPES buffer under shaking. Fluorescence intensity was recorded for cell autofluorescence (excitation at 640 nm, emission above 800 nm) and SWCNTs in confocal mode (excitation at 780 nm, emission above 980 nm).


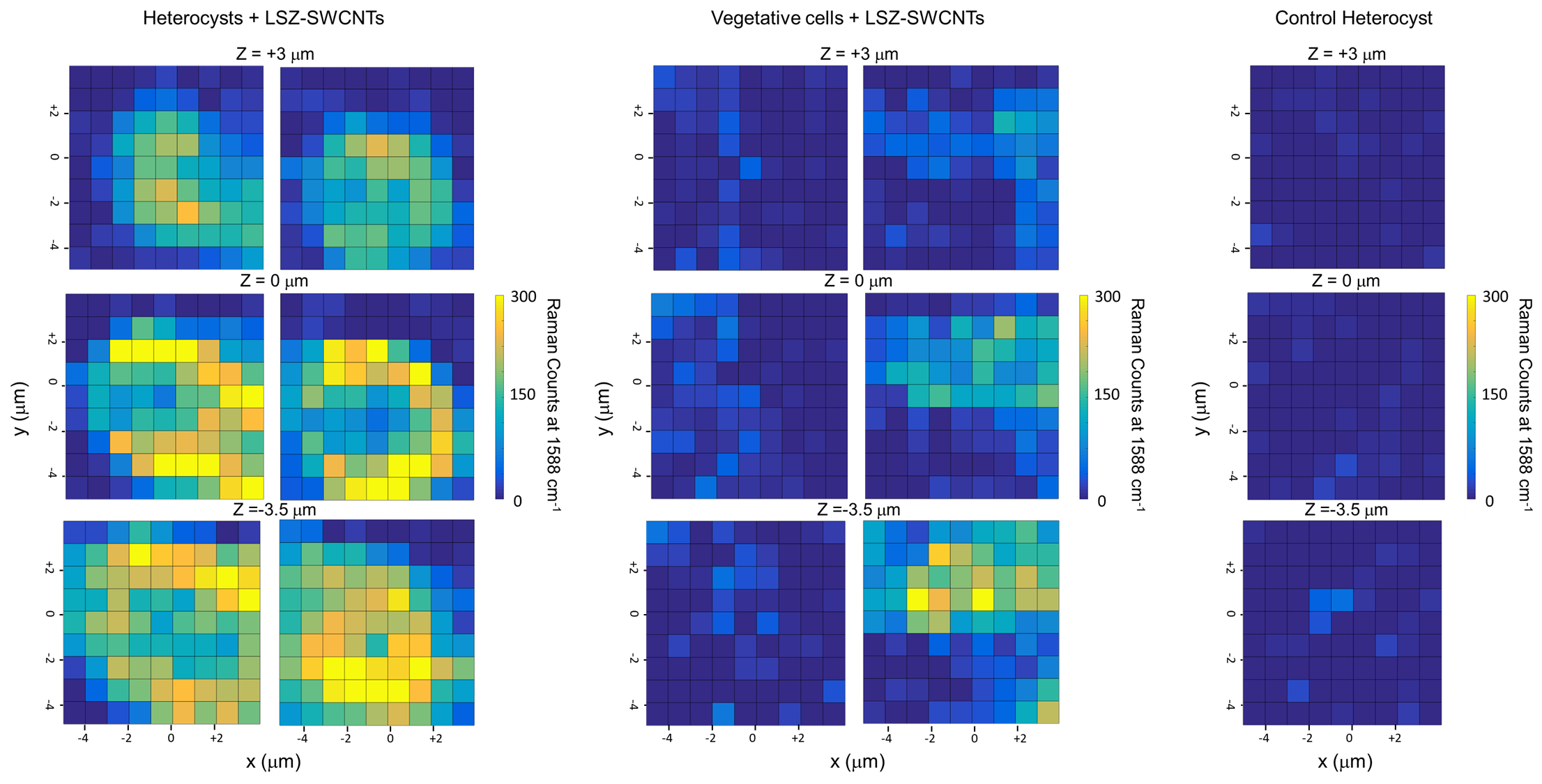


**Figure S4. Confocal Raman Characterization of SWCNT Distribution in *Nostoc* Heterocysts.** Representative confocal 3D Raman mapping of the characteristic SWCNT G’-band used to explore the spatial distribution of LSZ-SWCNTs within two heterocysts (left) or vegetative cells (center) of *Nostoc* filaments. The cells are immobilized onto a poly-lysine-coated glass slide. Values correspond to SWCNT intensity at 1580 cm^-1^ under 532 nm excitation. Confocal Raman z-stacks acquired for a *Nostoc* heterocyst without LSZ-SWCNTs (right) shows no contaminating signal at 1580 cm^-1^ under 532 nm excitation.


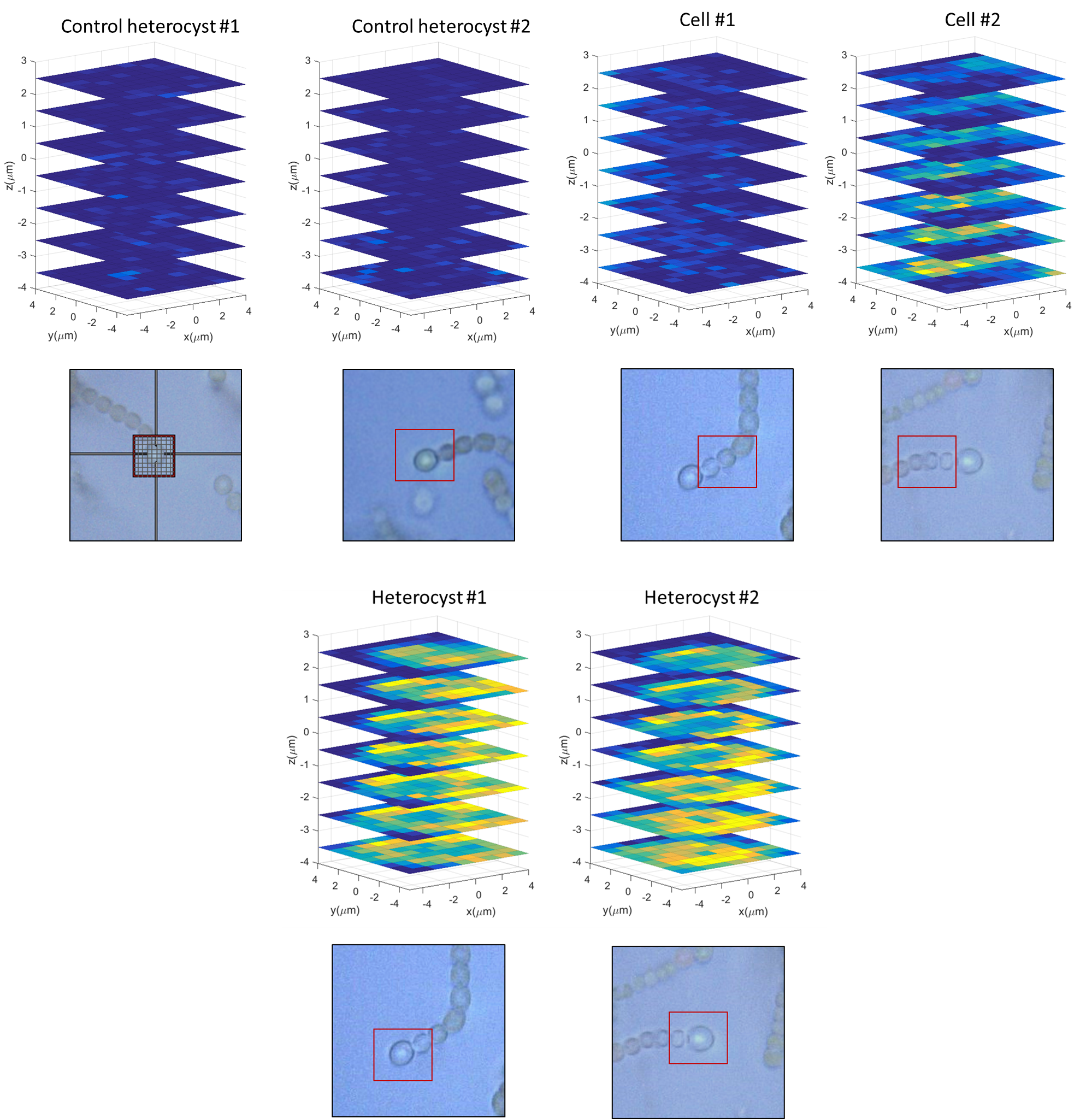


**Figure S5. 3D Confocal Raman Maps of SWCNT Distribution in *Nostoc* Heterocysts.** Full confocal Raman 3D maps of the characteristic SWCNT G’-band used to explore the spatial distribution of LSZ-SWCNTs within heterocysts (Heterocyst #1 and #2) or vegetative cells (Cell #1 and #2 centered in their respective frames) of *Nostoc* filaments. 3D Raman maps of the G’-band distribution in heterocysts in the absence of LSZ-SWCNTs are shown as a control (Control heterocyst #1 and #2). The images below each Raman map show the corresponding bright-field images of analyzed *Nostoc* cells. The first image shows a 9 x 9 mapping grid centered on a *Nostoc* heterocyst.


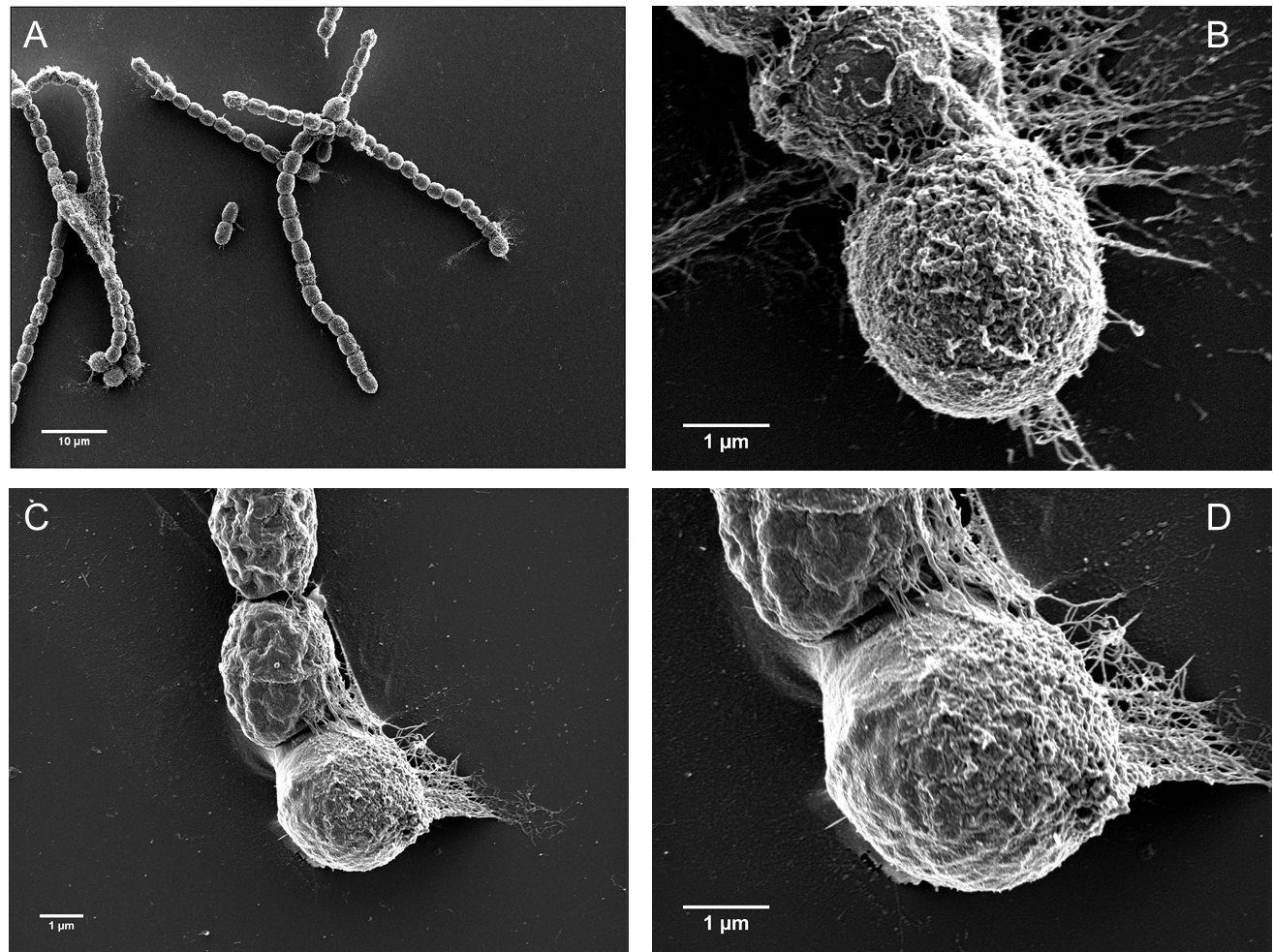


**Figure S6. Scanning Electron Microscopy (SEM) of *Nostoc*-Het Cells with LSZ-SWCNTs.** (a-d) SEM images of *Nostoc*-Het cells treated with LSZ-SWCNTs for (a,c) low magnification and (b,d) high magnification. The scale bars represent 1 µm, except for the panel (a) for which the scale represents 10 µm.


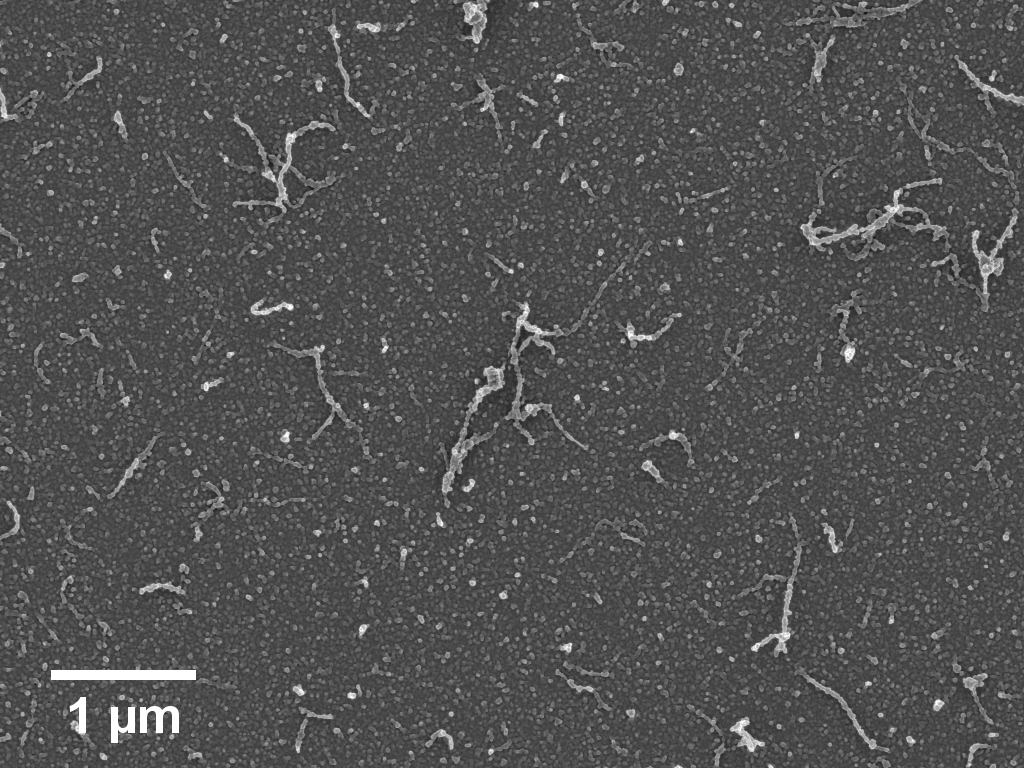


**Figure S7. Scanning electron microscopy (SEM) of LSZ-SWCNTs.** Representative SEM image of LSZ-SWCNTs immobilized onto a poly-lysine-coated glass slide.


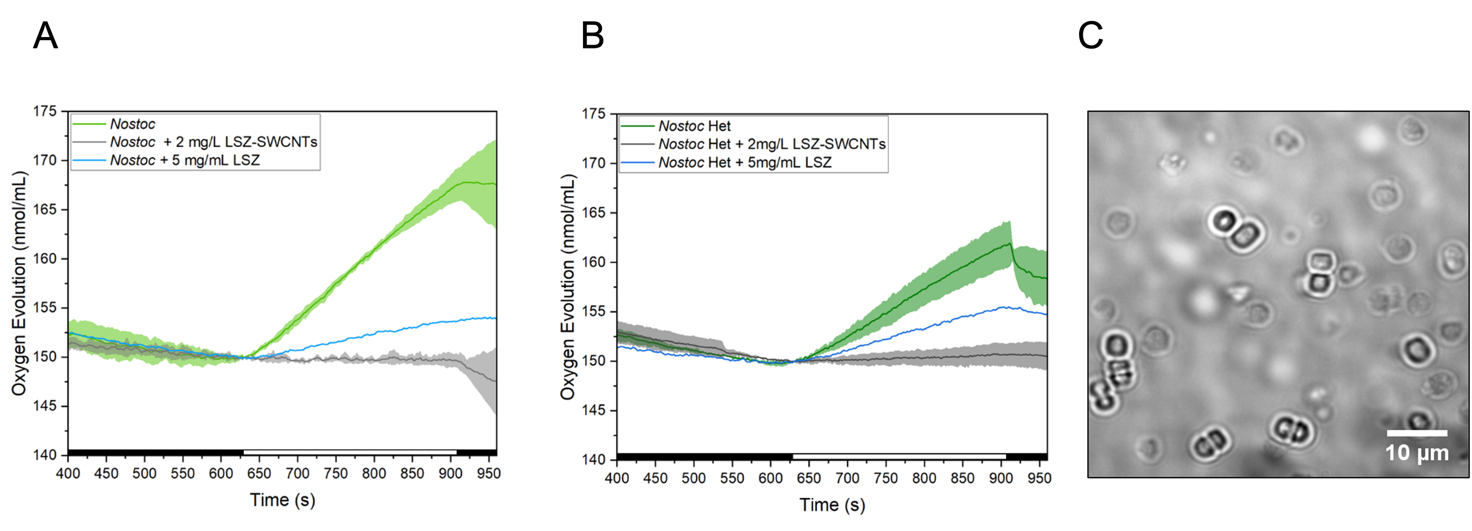


**Figure S8. Loss of Viability of *Nostoc* After Long Incubation with LSZ-SWCNTs.** Oxygen evolution measurements upon dark (black bar, x-axis) and light (white bar, x-axis) exposure to 100 μmol photons m^-2^s^-1^ for treated and untreated cells of *Nostoc* (A) and *Nostoc-*Het (B). Treatment includes incubation of the cells with either 2 mg/L LSZ-SWCNTs (triplicate measurements) or 5 mg/mL LSZ (single measurement) under the same conditions. The slope of evolved oxygen under light conditions corresponds to the oxygen evolution rate and indicates photosynthetic capacity and thereby viability. For triplicate measurements, the line corresponds to the mean value and the shading represents 1 standard deviation. (C) Representative bright-field image of *Nostoc* cells after prolonged incubation with 2 mg/L LSZ-SWCNTs. Almost complete rupture of the filaments is observed.


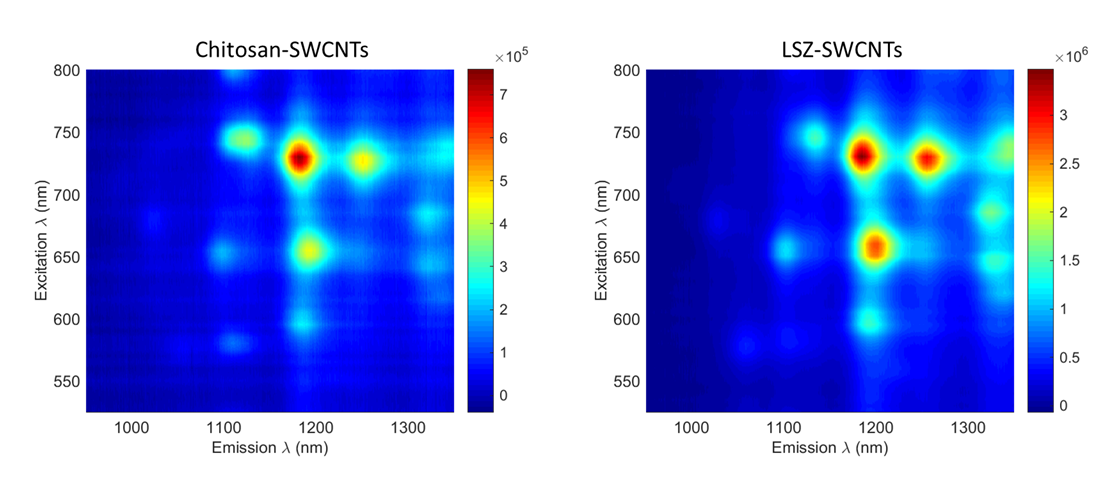


**Figure S9. Optical Characterization of Chitosan-SWCNTs and LSZ-SWCNTs Suspensions.** Photoluminescence plots of chitosan-SWCNTs (left) and LSZ-SWCNTs (right) at the same SWCNT concentration as determined by absorbance measurement at 632 nm.


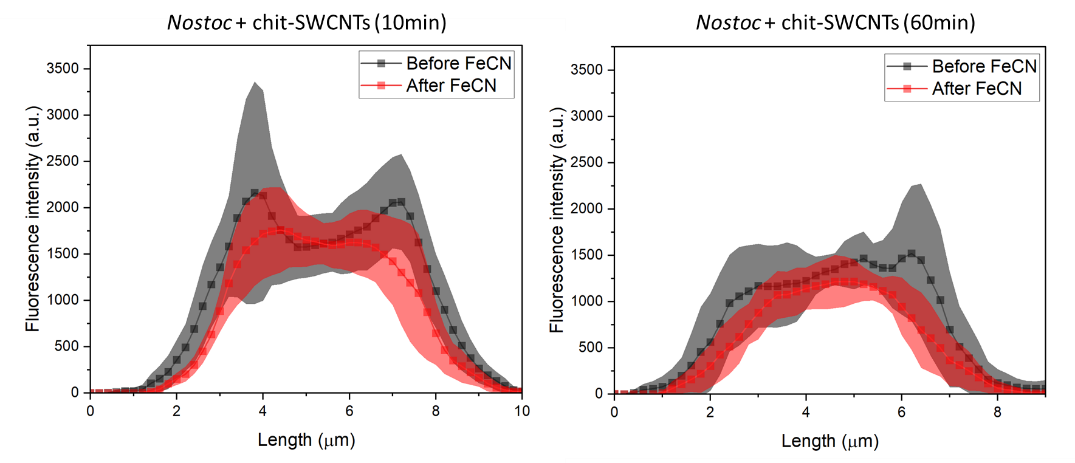


**Figure S10. Ferricyanide Quenching of Accessible SWCNTs.** SWCNT fluorescence along the cell diameter traced from *Nostoc* cells (n = 6) incubated with chitosan-SWCNTs for 10 minutes (left) or 60 minutes (right) before (black) and after (red) addition of 120 mM ferricyanide. The shaded regions represent a standard deviation of 1 σ.


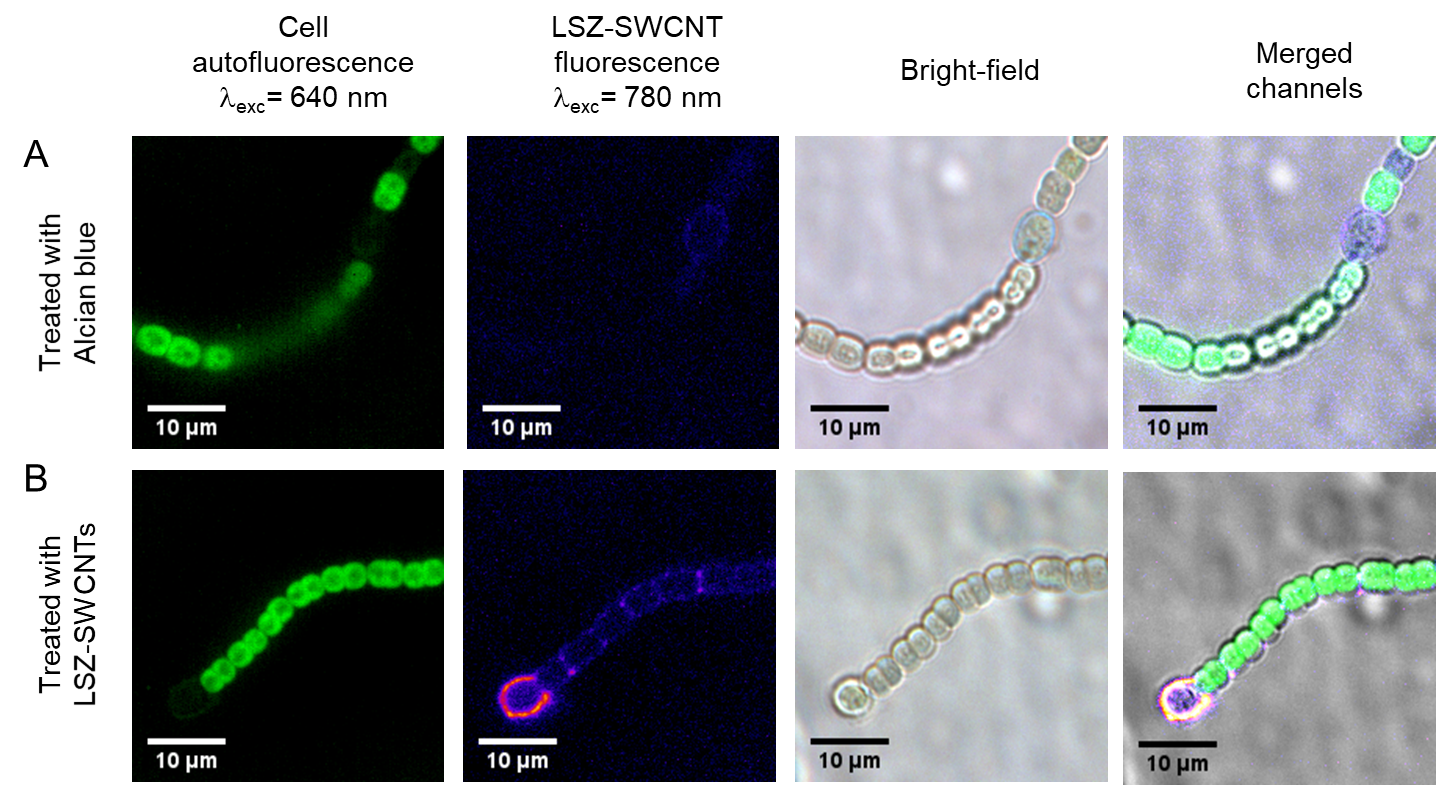
3

**Figure S11. NIR Imaging of Stained Heterocysts.** Representative images of *Nostoc*-Het cells stained with (A) Alcian Blue and (B) with LSZ-wrapped SWCNTs. Fluorescence intensity was recorded for cell autofluorescence (excitation at 640 nm, emission above 800 nm) and SWCNTs in confocal mode (excitation at 780 nm, emission above 980 nm).


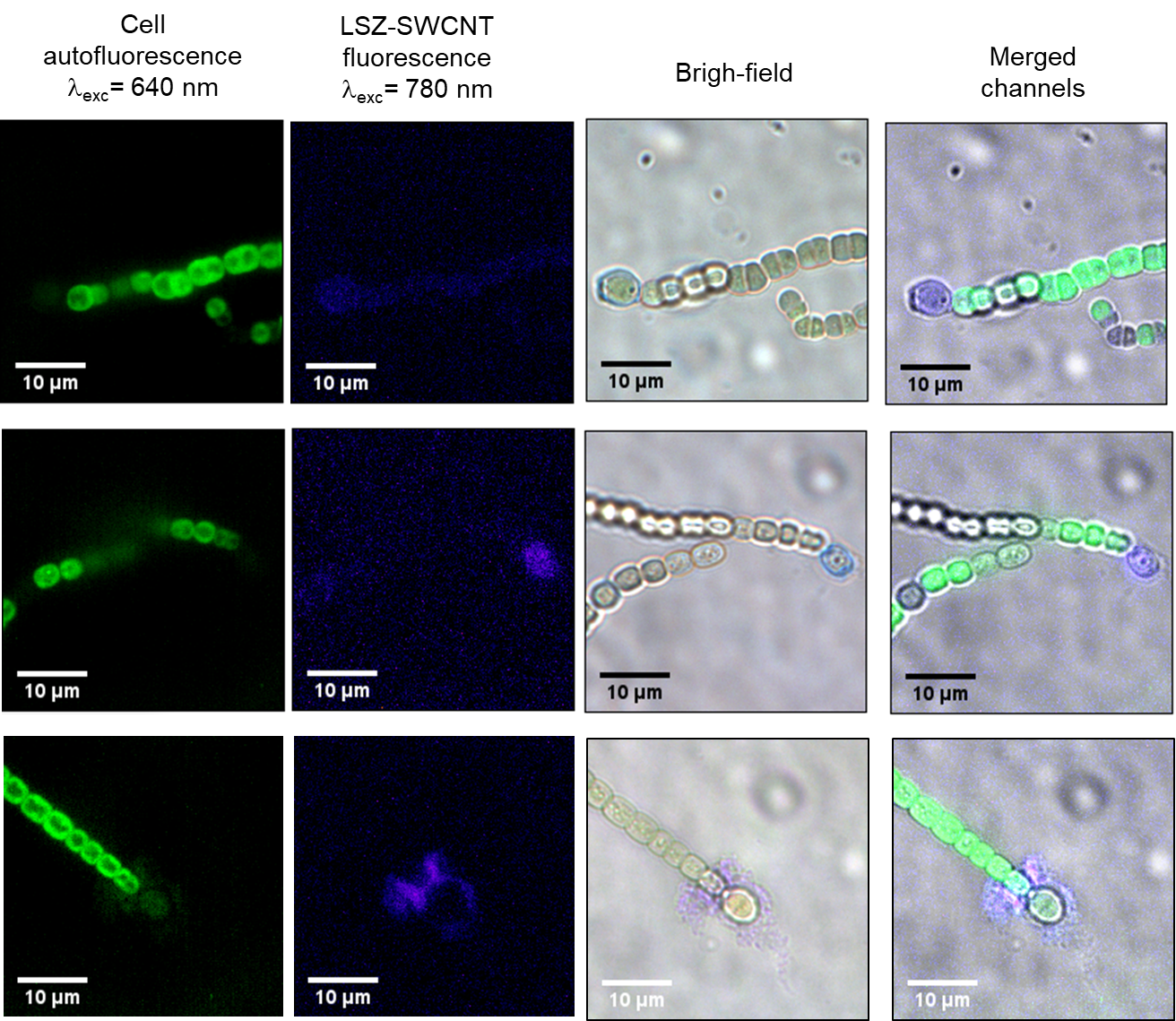


**Figure S12. Staining of *Nostoc* Heterocysts with Alcian Blue.** Representative images of *Nostoc*-Het cells immobilized onto poly-lysine-coated glass slides after incubation with Alcian blue. Fluorescence intensity was recorded for cell autofluorescence (excitation at 640 nm, emission above 800 nm) and SWCNTs in confocal mode (excitation at 780 nm, emission above 980 nm). Alcian blue stains the exo-polysaccharide in blue.

**References**

[*] Ermakova, M., Battchikova, N., Richaud, P., Leino, H., Kosourov, S., Isojarvi, J., Peltier, G., Flores, E., Cournac, L., Allahverdiyeva, Y. and Aro, E., 2014. Heterocyst-specific flavodiiron protein Flv3B enables oxic diazotrophic growth of the filamentous cyanobacterium Anabaena sp. PCC 7120. *Proceedings of the National Academy of Sciences*, 111(30), pp.11205-11210.
